# Effects of Parental Autonomy Support and Teacher Support on Middle School Students’ Homework Effort: Homework Autonomous Motivation as Mediator

**DOI:** 10.3389/fpsyg.2019.00612

**Published:** 2019-03-27

**Authors:** Xiaowei Feng, Ke Xie, Shaoying Gong, Lei Gao, Yang Cao

**Affiliations:** ^1^School of Psychology, Central China Normal University, Wuhan, China; ^2^Key Laboratory of Adolescent Cyberpsychology and Behavior, Ministry of Education, Wuhan, China

**Keywords:** homework effort, parental autonomy support, teacher support, homework autonomous motivation, mathematics, middle school students

## Abstract

The present study tested whether students’ autonomous motivation mediated the association between adult support (parental autonomy support, teacher support) and students’ homework effort. A sample of 666 Chinese middle school students was recruited to complete the parental autonomy support questionnaire, teacher support questionnaire, homework autonomous motivation questionnaire and homework effort questionnaire. Structural equation modeling showed that both parental autonomy support and teacher support positively predicted mathematics homework effort, and mathematics homework autonomous motivation was a mediator in these associations. The present study reveals the importance of adult support and autonomous motivation, and has theoretical and practical implications.

## Introduction

Homework refers to tasks assigned by teachers, which students complete during their extracurricular time (Cooper, 2001). Students’ effort toward homework is predictive of homework outcomes (Trautwein and Köller, 2003; Trautwein and Lüdtke, 2007). The homework model holds that homework effort is evidenced in four dimensions: investment, compliance (doing homework carefully and independently), concentration (doing homework with focus) and number of tasks (percentage of tasks attempted) (Trautwein et al., 2006). Furthermore, Trautwein et al. (2006) developed the Homework Effort Questionnaire with three subscales: homework completion compliance, concentration, and percentage of tasks attempted. Academic engagement research consistently connects homework effort with performance (Trautwein and Köller, 2003; Trautwein and Lüdtke, 2007; Flunger et al., 2015). Recent evidence suggests that homework effort is associated with environmental variables such as family and school factors, including homework quality (Dettmers et al., 2010; Liu et al., 2016), teacher feedback and support (Liu et al., 2017), parental involvement quality and motivation for homework (Dumont et al., 2014; Liu et al., 2017), and individual variables such as gender (Xu, 2011), conscientiousness (Trautwein et al., 2006; Flunger et al., 2017), homework motivation (Trautwein and Lüdtke, 2009; Flunger et al., 2017; Liu et al., 2017), and homework emotions (Dettmers et al., 2011; Goetz et al., 2012; Liu et al., 2017). This study concerned how adult support from family and school influences homework together. Existing studies have found that adult support is positively associated with adolescents’ social and academic adjustment (Kocayörük et al., 2015; Tennant et al., 2015; Morton, 2016). However, it is still unclear how parent support and teacher support together impact homework effort.

Based on the self-determination theory, the satisfaction of adolescents’ needs-autonomy, competence and relatedness-is fundamental to autonomous motivation (Deci and Ryan, 2000), leading to higher academic engagement (Roth et al., 2009). Previous studies explored the relationship between parental involvement and adolescents’ academic engagement and performance (Moon and Hofferth, 2016; Boonk et al., 2018). The present study extends and expands literature by considering the concurrent influences of parental support and teacher support on homework effort. Accordingly, we expected that parental autonomy support and teacher support positively predict homework effort. In addition, adult support, as an external factor, affects individual behaviors via internal factors (Helgeson and Lopez, 2010). Therefore, the present study also tested the mediating role of autonomous motivation.

### Parental Autonomy Support and Homework Effort

Previous studies have found that parental involvement which includes parental autonomy support as one indicator is closed to learning (Cheung and Pomerantz, 2011; Choi et al., 2015). Parental involvement means parents involve in children’s schooling to contribute to their academic achievement (Cheung and Pomerantz, 2011; Baker, 2015). In general, parental involvement is significantly predictive of students’ academic performance (Choi et al., 2015; Moon and Hofferth, 2016). A meta-analysis study found that parental homework involvement was significantly associated with students’ general achievement and mathematics achievement, though the effect sizes were very small (ES = 0.024; ES = 0.063) (Castro et al., 2015). However, parents’ content support, one form of parental support, is not always positively, even negatively, related to students’ academic performance (including mathematics performance); parents’ controlling or intrusive support impairs adolescents’ motivation and academic achievement (Levpuscek and Zupancic, 2009; Boonk et al., 2018; Xu et al., 2018). Specifically, research in parental involvement in homework, however, has found that parental involvement in homework both promoted and impaired students’ performance (Desimone, 1999; Cooper et al., 2000; Corno and Xu, 2004; Pomerantz et al., 2005). Some researchers hold that the quality rather than the quantity of parental involvement in homework is crucial to students’ achievement (Trautwein et al., 2006; Knollmann and Wild, 2007), and revealed some dimensions that positively associate with academic engagement and achievement, for example, parental autonomy support (Cooper et al., 2000; Pomerantz et al., 2007; Katz et al., 2011; Dumont et al., 2012; Moroni et al., 2015; Boonk et al., 2018; Xu et al., 2018).

Parental autonomy support is critical to adolescents’ development and learning. Parental autonomy support can be defined as parental encouragement of students’ problem-solving, selection and decision-making (Grolnick, 1989). Parental autonomy support is demonstrated through honoring students’ opinions, encouraging self-determination, providing opportunities to make independent choices, avoiding the use of controlling language, and offering an autonomous family environment (Deci and Ryan, 2012). A family environment that supports autonomy encourages adolescents to solve problems actively, think independently and search for an identity, thus improving their sense of control and competence.

Extensive research has shown that parents’ support for autonomy is beneficial to students’ learning engagement (Roth et al., 2009), academic ability and achievement (Soenens and Vansteenkiste, 2005; Wang et al., 2007; Liew et al., 2014; Pomerantz et al., 2014; Vasquez et al., 2016). A qualitative study of 15 parents showed that parent-reported support for autonomy was associated with students’ homework enjoyment (Froiland, 2015). Froiland (2011) intervened to improve parental autonomy support for 7 weeks, which improved elementary school students’ positive emotions about homework and their academic intrinsic motivation. Further, a study of elementary and junior high school students showed that parents’ support for autonomy in homework was beneficial for students’ mastery goals and achievement (Gonida and Cortina, 2014).

According to the self-determination theory (Deci and Ryan, 2000), adolescents urge parental support for autonomy, for example, being encouraged to arrange homework and solve problems independently. Such support is beneficial to students’ autonomous motivation and learning engagement (Roth et al., 2009), academic performance (Boonk et al., 2018; Xu et al., 2018), and healthy development and well-being (Kocayörük et al., 2015). Based on the previous work, the present study proposed that parental autonomy support is associated with students’ homework effort positively.

### Teacher Support and Homework Effort

As leaders of class activities, teachers are frequently in contact with students and are important sources of support. The types of teacher support perceived by students in school include autonomy support, cognitive support and emotional support (Chai et al., 2011; Chai and Gong, 2013). Autonomy support is indicated by teachers’ respect for students’ opinions and feelings, opportunities to choose, encouragement of independent problem-solving, and provision of time for thinking (Lam et al., 2009; Chai et al., 2011; Chai and Gong, 2013). Teachers’ cognitive support is demonstrated through providing students learning strategies, guiding the problem-solving processes, and offering reasonable assignments and effective feedback (Chai et al., 2011). Emotional support concerns teachers’ care for their students, connection to students’ emotions, and response to students’ negative academic emotions (Rosiek, 2003; Titsworth et al., 2010).

Existing research has shown that teacher support is linked to students’ mastery motivation (Ruzek et al., 2016), positive emotions, the use of self-regulated strategies (Wang and Eccles, 2013), academic engagement (Jang et al., 2010, 2016) and academic effort (Federici and Skaalvik, 2014). In mathematics domain, Sakiz et al. (2012) found that perceived teacher support was positively associated with middle school students’ self-efficacy beliefs, academic enjoyment and academic effort. In a sample of Chinese elementary school students, Liu et al. (2018) found that teacher support directly affected students’ mathematics engagement in cognitive, behavioral and emotional realms. Taken together, it was hypothesized that students’ homework effort is impacted by teacher support.

### The Mediating Role of Autonomous Motivation

Autonomous motivation is an individual factor that may mediate the associations between adult support and homework effort. Autonomous motivation refers to the motivation that individuals experience when they have volition and free choice. For instance, interest is a typical autonomous motivation (Deci and Ryan, 2000). Autonomous motivation takes on great significance in individuals’ learning behaviors; for example, it significantly predicts junior high school students’ academic effort and achievement (Mih, 2013; Mouratidis et al., 2018). Similarly, homework motivation, which activates students in doing homework, is critical to homework achievement (Ayten and Eunsook, 2012).

Parents’ and teachers’ supportive behaviors promote the internalization of students’ learning motivation, thus activating their autonomous motivation (Grolnick et al., 2007; Roth et al., 2007; Froiland, 2011). Extensive research has shown that parental autonomy support promotes students’ academic autonomous motivation (Froiland, 2015; Vasquez et al., 2016); teacher support also significantly predicts elementary and middle school students’ intrinsic motivation and autonomous motivation for homework (Katz et al., 2009; Hagger et al., 2015; Liu et al., 2017). In addition, autonomous motivation research connects parent and teacher support with students’ academic effort and achievement (Mih, 2013). However, it is still unknown whether autonomous motivation for homework is a mediator in the relationships between adult support and students’ homework effort.

### The Present Study and Hypotheses

Given that homework effort is subject-specific (Trautwein et al., 2006), we focused on mathematics, which is the basic subject of STEM disciplines (English, 2016). Compared to mathematics in primary grades, mathematics in middle school increases in content and complexity, and thus middle school students may need more support from adults, such as teachers and parents (Zhang, 2016). Adult support can be measured by different methods such as direct observation or self-report from adults or students. However, studies focusing on adult support provide complex results. On the one hand, researchers found that students’ perceived supportive teaching is positively associated with students’ motivation and engagement (Stroet et al., 2013; Patall et al., 2018). On the other hand, compared to students’ perceived teacher support, directly observed or teacher-reported supportive teaching has smaller or little association with students’ motivation and engagement (Stroet et al., 2013). Therefore, students’ perceived teacher support rather than teacher self-reported support or observed teacher support was considered as an important adult support in the present study.

Next, according to expectancy-value theory of achievement motivation, parental support reported by parents is the more distal factor, while parental support reported by students is the more proximal factor (Wigfield and Eccles, 2000). Parents-reported support influences students’ achievement motivation through students’ perceived parental support (Dinkelmann and Buff, 2016). As a consequence, students’ perceived support may have a stronger association with their achievement. In the current study, students’ perceptions of parental autonomy support and teacher support were measured.

The present study explored the effect of adult support on homework and tested the following hypotheses. We tested the effects of parental autonomy support and mathematics teacher support on middle school students’ homework effort. (H1a) Middle school students’ perceptions of parental autonomy support and of (H1b) mathematics teacher support will positively predict mathematics homework effort. The mediating role of mathematics autonomous motivation in the links between parental autonomy support and mathematics homework effort, and between mathematics teacher support and mathematics homework effort, was also tested. (H2a) Parental autonomy support and (H2b) mathematics teacher support will predict mathematics homework effort through mathematics autonomous motivation.

## Materials and Methods

### Participants

Participants were 666 seventh and eighth graders recruited from three middle schools in the cities of Wuhan and Xiaogan in Hubei Province, Central China. All the schools were in middle to upper middle level in the two cities. All the classes were randomly selected from the schools. Of these, 322 were seventh graders (169 males) from seven different classes and 344 were eighth graders (182 males) from eight different classes. Their average age was 12.91 years (*SD* = 0.78).

### Measures

#### Parental Autonomy Support

Parental autonomy support was assessed by the Psychological Autonomy Support Questionnaire, a Chinese-language measure developed by Wang et al. (2007). The questionnaire consists of eight items, including two subscales which assess choice making (4 items, e.g., “My parents allow me to make choices whenever possible”) and opinion exchange (4 items, e.g., “My parents encourage me to give my ideas and opinions when it comes to decisions about me”). Each item was rated on a scale from 1 (not at all true) to 5 (very true). Higher scores indicate greater parental autonomy support. In the current study, CFA results showed that: χ^2^/*df* = 3.21, RMSEA = 0.06, SRMR = 0.03, TLI = 0.96, CFI = 0.98. The overall score (the mean of 8 items) was used in the current study, with α = 0.88.

#### Mathematics Teacher Support

Mathematics teacher support was measured with the Questionnaire on Perceived Mathematics Teacher Support for Middle School Students, a Chinese-language measure developed by Chai and Gong (2013). This questionnaire consisted of three subscales: teacher autonomy support (5 items, e.g., “When solving mathematics problems, the mathematics teacher allows us to propose solutions that differ from the standard answers”), teacher cognitive support (5 items, e.g., “The mathematics teacher encourages us to look for solutions rather than telling us the answers directly”), and teacher affective support (7 items, e.g., “The mathematics teacher knows and cares about me”). All items were rated on a scale from 1 (not at all true) to 5 (very true). Higher scores indicate greater teacher support. In the present study, CFA results showed that: χ^2^/*df* = 3.26, RMSEA = 0.06, SRMR = 0.05, TLI = 0.93, CFI = 0.94. The overall score (the mean of 17 items) was used in the current analyses. The Cronnbach’s α in this study is 0.94.

#### Mathematics Homework Autonomous Motivation

To assess students’ mathematics autonomous motivation, we used the Chinese version of the Questionnaire on Students’ Autonomous Motivation in Mathematics Homework (Liu et al., 2017; for original version, see Katz et al., 2011). The original questionnaire consisted of an autonomous motivation subscale (11 items, e.g., “I do my homework because it is fun”) and controlled motivation subscale (8 items). The present study used the autonomous subscale, with items rated on a scale from 1 (not at all true) to 5 (very true). Five items were deleted because their factor loadings were lower than 0.5 in CFA results (Hair et al., 1998). The resulting 6-item scale had α = 0.89. In the current study, CFA results showed that: χ^2^/*df* = 3.52, RMSEA = 0.06, SRMR = 0.03, TLI = 0.98, CFI = 0.99.

#### Homework Effort

The Chinese version of the Homework Effort Questionnaire was used to assess homework effort (Zhang, 2008; for original version, see Trautwein et al., 2006). This 8-item questionnaire included three dimensions: homework completion compliance (3 items, e.g., “I’ve recently been doing my mathematics homework to the best of my ability”), concentration (4 items, e.g., “I concentrate hard when I do my mathematics homework”), and percentage of tasks attempted. Items were rated on a 4-point Likert scale from *extremely disagree* to *extremely agree*. The measure has been shown to be reliable in mathematics research, with α = 0.81 (Liu et al., 2016). To fit domain specificity, we added the word “mathematics” before “homework” in each item. In the current study, CFA results showed that: χ^2^/*df* = 3.64, RMSEA = 0.06, SRMR = 0.04, TLI = 0.92, CFI = 0.96. The Cronnbach’s α in this study is 0.78.

### Procedure

The study was first approved by the Ethical Committee of the author’s University. Then, informed written consent was provided by all middle schoolers, parents’ written informed consent was obtained separately before the assessment. After that, paper-and-pencil questionnaires were group-administered to middle school students during regular class sessions by trained graduate students who were major in psychology. All the graduate students administered the assessment according to the same guidelines. The whole process took about 20 min.

### Data Analysis

First, Pearson correlations were used to test relationships among variables. Next, the measurement model was set up with Mplus 7 to assess whether indicators represented their latent variables, and to examine correlations among all latent variables. We used χ^2^/*df*, RMSEA, SRMR, CFI, and TLI to assess model fit. For RMSEA, a value ≤0.05 represents good model fit, and 0.08 is acceptable (Browne and Cudeck, 1993); for SRMR, a value <0.08 is acceptable (Hu and Bentler, 1998); A CFI value ≥0.90 or a TLI value ≥0.90 represent an acceptable model fit (Hu and Bentler, 1999; Byrne, 2010).

We set up a structural equation model to test our hypotheses. Considering the hierarchical structure of the data, we used “type = complex” (in the analysis command) and “cluster = class” (in the variable command) to compute the standard errors and chi-square tests of model fit. The maximum likelihood estimation in Mplus 7 was also selected. To reduce the complexity of the model, we used item parceling with dimensional scales as item parcels (Bandalos, 2002), but homework autonomous motivation was parceled as three item parcels according to its single-factor CFA results. We first tested the predictions regarding parental autonomy support and teacher support as predictors of homework effort. Second, the mediating role of homework autonomous motivation in each link between support and effort was examined. We used the indexes χ^2^/*df*, RMSEA, SRMR, CFI, and TLI to assess model fit. According to Rosenthal and Rosnow (1991), we used Cohen’s *d* to report the sizes of the effects and indirect effects. Finally, we used bootstrapping and an estimated bias-corrected 95% confidence interval to test the indirect effects.

## Results

### Preliminary Analyses

The descriptive statistics and Pearson correlations among the variables are presented in Table 1. The correlations among all of the variables are significant. We examined the distributions of variables with skewness and kurtosis, and the results showed that all the variables are normal distributions according to the criterion of Finney and DiStefano (2006) (see Table 1).

**Table 1:** Descriptive statistics and Pearson correlations among variables.

	1	2	3	4	5	6	7	8	9	10	11
1 PAS1	—										
2 PAS2	0.76^∗∗^	—									
3 TS1	0.20^∗∗^	0.20^∗∗^	—								
4 TS2	0.14^∗∗^	0.18^∗∗^	0.75^∗∗^	—							
5 TS3	0.11^∗∗^	0.15^∗∗^	0.70^∗∗^	0.84^∗∗^	—						
6 HAM1	0.24^∗∗^	0.28^∗∗^	0.37^∗∗^	0.32^∗∗^	0.28^∗∗^	—					
7 HAM2	0.26^∗∗^	0.29^∗∗^	0.44^∗∗^	0.40^∗∗^	0.31^∗∗^	0.73^∗∗^	—				
8 HAM3	0.28^∗∗^	0.27^∗∗^	0.41^∗∗^	0.34^∗∗^	0.27^∗∗^	0.71^∗∗^	0.78^∗∗^	—			
9 HE1	0.25^∗∗^	0.23^∗∗^	0.26^∗∗^	0.19^∗∗^	0.16^∗∗^	0.28^∗∗^	0.35^∗∗^	0.37^∗∗^	—		
10 HE2	0.24^∗∗^	0.21^∗∗^	0.27^∗∗^	0.14^∗∗^	0.14^∗∗^	0.37^∗∗^	0.40^∗∗^	0.39^∗∗^	0.36^∗∗^	—	
11 HE3	0.13^∗∗^	0.13^∗∗^	0.30^∗∗^	0.21^∗∗^	0.20^∗∗^	0.22^∗∗^	0.24^∗∗^	0.29^∗∗^	0.34^∗∗^	0.32^∗∗^	—
*M*	3.63	3.69	3.57	4.07	4.23	3.53	3.67	3.43	3.53	2.67	3.62
*S*	1.06	1.06	1.03	0.90	0.89	1.22	1.16	1.23	0.53	0.50	0.66
*Skewness*	-0.58	-0.68	-0.57	-1.20	-1.53	-0.45	-0.56	-0.42	-1.56	-0.30	-1.99
*Kurtosis*	-0.49	-0.31	-0.25	1.33	2.15	-0.79	-0.62	-0.80	3.29	-0.36	4.15

*PAS1, PAS2 (Measures of Parental autonomy support), TS1, TS2, TS3 (Measures of Teacher support), HAM1, HAM2, HAM3 (Measures of Homework autonomous motivation), HE1, HE2, HE3 (Measures of Homework effort). *N* = 666, ^∗∗^*p* < 0.01*.

Follow-up difference tests indicated that boys reported higher homework autonomous motivation than girls, *M*_Male_ = 3.67, *M*_Female_ = 3.38, *t* = 3.57, *p* < 0.001, *d* = 0.34. Therefore, we controlled the impacts of gender on homework autonomous motivation.

### Measurement Model

The measurement model included four latent factors (parental autonomy support, teacher support, homework autonomous motivation, and homework effort) and 11 observed variables. An initial test of the measurement model indicated a good fit to the data, χ^2^/*df* = 3.06, RMSEA = 0.06, SRMR = 0.05, CFI = 0.96, and TLI = 0.95. Most loadings were higher than 0.80, the lowest standardized loading being 0.49 for a homework effort item. This indicated that the measurement model had sufficient convergent validity.

### The Mediating Role of Mathematics Homework Autonomous Motivation

According to our hypotheses and Pearson correlations results, we conducted structural equation model analysis with parental autonomy support and mathematics teacher support as predictors, homework autonomous motivation as mediator, and mathematics homework effort as outcome variable. Gender was also included as a covariate for homework autonomous motivation. Following the suggestions from Bandalos (2002), item parceling was used to reduce the complexity of the model, with dimensional scales as item parcels. Mathematics homework autonomous motivation as a single dimension scale was created using single-factor CFA before item parceling. Results demonstrated that the model fit the data well (χ^2^/*df* = 4.55, RMSEA = 0.07, SRMR = 0.07, CFI = 0.97, TLI = 0.98), so we parceled mathematics homework autonomous motivation as three item parcels. According to the mediating effect analysis procedure (Wen and Ye, 2014), we first analyzed the direct roles of parental autonomy support and teacher support on mathematics homework effort. Results showed that parental autonomy support (*b* = 0.35, *p* < 0.001, *d* = 0.52) and teacher support (*b* = 0.31, *p* < 0.001, *d* = 0.49) significantly predicted mathematics homework effort.

**Figure 1:**
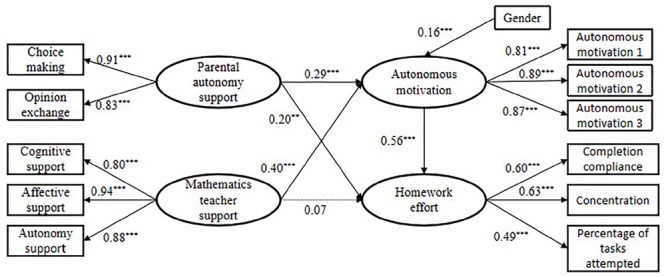
Structural model of the associations among parental autonomy support, teacher support, autonomous motivation and homework effort. ^∗^*p* < 0.05, ^∗∗^*p* < 0.01, ^∗∗∗^*p* < 0.001.

The present study tested the mediating role of mathematics homework autonomous motivation in the relations between parental autonomy support and mathematics homework effort, and between mathematics teacher support and mathematics homework effort (see Figure 1). This model indicated a good fit to the data (χ^2^/*df* = 2.86, RMSEA = 0. 05, SRMR = 0.06, TLI = 0. 96, CFI = 0.97). Results showed that parental autonomy support and mathematics teacher support both positively predicted mathematics homework autonomous motivation (*b* = 0.29, *p* < 0.001, *d* = 0.58; *b* = 0.40, *p* < 0.001, *d* = 0.82). In addition, mathematics homework autonomous motivation significantly predicted mathematics homework effort (*b* = 0.56, *p* < 0.001, *d* = 0.73). Then, adding the mediating variable (mathematics homework autonomous motivation), parental autonomy support still significantly predicted mathematics homework effort (*b* = 0.20, *p* < 0.001, *d* = 0.30), however, mathematics teacher support was no longer predictive of mathematics homework effort (*b* = 0.07, *p* > 0.05). This result indicated that mathematics homework autonomous motivation partly mediated the relationship between parental autonomy support and mathematics homework effort, and fully mediated the relationship between mathematics teacher support and mathematics homework effort.

Finally, we used bootstrapping to test whether the above mediating effects were significant (Preacher and Hayes, 2008). Results indicated that the 95% confidence interval of the mediating effect on the association between parental autonomy support and mathematics homework effort was [0.091, 0.266], and the 95% confidence interval of the mediating effect on the relationship between mathematics teacher support and mathematics homework effort was [0.154, 0.311]. These two 95% confidence intervals did not include 0 (see Table 2), suggesting that the mediating role of mathematics homework autonomous motivation was significant in both cases.

**Table 2:** Path coefficients of the model.

Path	Estimated effect	SE	95% CI	*p*	Cohen’s *d*
PAS → HAM → HE	0.164	0.043	[0.091, 0.266]	<0.001	0.29
TS → HAM → HE	0.224	0.040	[0.154, 0.311]	<0.001	0.44

*PAS, parental autonomy support; HAM, homework autonomous motivation; HE, homework effort; TS, teacher support*.

## Discussion

It is difficult to overstate the importance of homework (Fan et al., 2017), a key element of which is homework effort. Building on previous research on homework effort, we tested the effects of students’ perceived parental autonomy support and teacher support on middle school students’ mathematics homework effort. Our results showed that both parental autonomy support and teacher support perceived by middle school students positively predicted mathematics homework effort, and mathematics homework autonomous motivation was a mediator in these associations. These findings suggest that support from parents and teachers is beneficial to middle school students’ autonomous motivation and homework effort.

### The Effect of Parental Autonomy Support on Mathematics Homework Effort

As expected, the present study found that students’ perceived parental autonomy support positively predicted middle school students’ homework effort. This means that students who perceived more parental autonomy support put greater effort into mathematics homework. A long line of studies has suggested that parental autonomy support enhances students’ academic engagement (Wang et al., 2007; Woolley and Bowen, 2007; Wang and Eccles, 2012). The present study found the same positive relationship between parental autonomy support and students’ homework effort, which supports the self-determination theory (Deci and Ryan, 2000). This theory holds that autonomy support influences individuals’ engagement in tasks by satisfying their need for autonomy. The sense of autonomy is a vital developmental task for adolescents (Van Petegem et al., 2012). As they reach puberty, young adolescents’ desire for autonomy seemingly comes out of the blue. With autonomy support from parents (e.g., encouragement to think independently and search for an identity), adolescents put more effort into learning tasks, increasing their sense of control.

### The Effect of Mathematics Teacher Support on Mathematics Homework Effort

The present results revealed that mathematics teacher support students perceived positively predicted middle school students’ homework effort, which confirmed Hypothesis 1b. Empirical studies have proven that teacher support significantly predicts students’ motivation, academic engagement and effort (Meyer and Turner, 2007; Jang et al., 2010; Federici and Skaalvik, 2014; Chai and Gong, 2015; Chen et al., 2015). Dietrich et al. (2015) also found that perceived teacher support is linked to intrinsic value and effort. In line with the above studies, we also found that students’ perceived teacher support positively impacted on homework effort. From the perspective of self-determination theory, autonomy support, cognitive support and emotional support provided by teachers meet students’ basic psychological needs, leading to a boost in their homework effort.

### The Mediating Role of Mathematics Homework Autonomous Motivation

As expected, the results from the present study revealed that mathematics homework autonomous motivation partially accounted for the relationship between students’ perceived parental autonomy support and mathematics homework effort. To be specific, perceived parental autonomy support appears to facilitate students’ autonomous motivation, and thus enhance effort in homework; at the same time, parental autonomy support could promote homework effort directly. A meta-analysis reported the positive relationship between parental autonomy support and students’ academic autonomous motivation (Vasquez et al., 2016); in addition, academic autonomous motivation significantly predicted academic engagement (Dong and Liu, 2016). By replicating earlier results showing that autonomous motivation is a mediator in the link between parental autonomy support and students’ effort (Mih, 2013), our results added evidence in the mathematics homework domain. Our results confirmed the self-determination theory (Deci and Ryan, 2000). That is, autonomy support strengthens students’ sense of control and competence by providing them meaningful opportunities to search for an identity. Consequently, growing up with autonomy support from parents, adolescents whose autonomy needs are satisfied are motivated to invest more effort in homework.

In line with previous studies (Chen et al., 2015), we found that the association between mathematics teacher support students perceived and students’ homework effort was mediated by homework autonomous motivation, which confirmed Hypothesis 2b. After adding homework autonomous motivation, the direct effect of teacher support was no longer significant. This means that support from mathematics teachers significantly promotes students’ mathematics homework autonomous motivation, thus increasing their homework effort. Once teachers provide autonomy support, it is likely to enhance students’ need for autonomy (Chen et al., 2015), intrinsic or autonomous motivation (Roth et al., 2007; Liu et al., 2017). Perceived teachers’ emotional support benefits teacher-student relationships, brings students a sense of belonging, and creates a willingness to consent to doing homework; this internalization of the value of homework leads to higher autonomous motivation and homework effort. Teacher cognitive support facilitates students’ learning motivation (Lam et al., 2009), which boosts their engagement in homework. Therefore, by satisfying students’ basic psychological needs, mathematics teacher support leads to students investing more effort in mathematics homework through enhancing their homework autonomous motivation.

Further, this study found gender difference in mathematics homework autonomous motivation. Existing studies have found that longitudinal changes of motivation do vary with gender (Lee and Kim, 2014). Furthermore, gender difference in motivation may increase in specific subject, such as mathematics. A large body of studies has reported that boys has higher intrinsic motivation than girls in mathematics (Meece et al., 2006; Huang et al., 2008; Lee and Kim, 2014). This study added new evidence of gender difference in mathematics homework domain. However, the gender differences in other variables were non-significant. This means that parents and teachers provided similar support for boys and girls, and the gender difference in mathematics homework autonomous motivation did not influence their mathematics homework effort significantly. Future research needs to consider the possible effect of gender on homework motivation and its role in the link between adult support and homework effort by enlarging sample and including other grades.

### Implications and Future Research

Theoretically, we provide empirical support for the self-determination theory in mathematics homework. Practically, our results also have implications for middle school students’ education in the family and school contexts. For the sake of middle school students’ mathematics learning, including mathematics homework effort, parents and teachers should provide more support, especially autonomy support. Parents can provide autonomy support from three perspectives: empathy, meaningful rationale and meaningful choices (Brenning et al., 2015). To be more specific, try to understand children’s perspective when communicate homework and school life; offer meaningful reasons why homework is important; allow children to arrange their homework time. These three key strategies also apply to teachers, for example, assigning tasks with different difficulty for students with different mathematics abilities, allowing them to solve problems with various strategies, providing support and feedback when they have confusion or problems, and encouraging and comforting students who are upset. Increased support from important others is beneficial to students’ autonomous motivation, and leads to effort and engagement in mathematics learning inclusive of mathematics homework.

The present study makes contributions to promoting mathematics homework effort; however, several limitations should be noted. Firstly, a cross-sectional design was used in this study, so the results fail to show causal relationships among variables. For instance, teachers may exhibit more supportive behaviors to students with high motivation and engagement (Nurmi, 2012); however, low-achieving students are more likely to be exposed to intrusive and controlling behaviors of teachers and parents (Nurmi, 2012; Su et al., 2015). Future research should consider using longitudinal methods, repeatedly measuring teacher and parent support, and students’ autonomous motivation and homework effort, to further test possible causal mechanisms affecting homework effort.

Secondly, although our investigation considered the effect of different adult support and mathematical autonomous motivation on mathematical homework effort by using structural equation model, all the measures were assessed by self-report. Future research needs to include multiple sources, for example, adults’ and students’ reports or observation to reveal the effect of adults’ support from different perspective on students’ mathematical homework effort. At the same time, the present study measured the effects of general parental autonomy support and mathematics teacher support on students’ homework effort. However, it is still unknown whether the effect sizes would be the same if the adult support is measured specific to homework. Studies on parental support specific to homework have found inconsistent results. For example, Dumont et al. (2012) found that parental homework support is positively associated with students’ academic achievement. While, parental homework support may also contribute to students’ helpless behaviors (Orkin et al., 2017). In the future, researchers can measure adult support specific to homework to explore its effects on homework effort and achievement.

Thirdly, the level of support from different teachers is different, so the ideal way is to construct multilevel structure model by incorporating teacher support as a teacher-level variable. However, we did not construct the multilevel model because of the small sample, so we cannot explain the relationship between teacher support and homework effort in class level. In the future, it is necessary to enlarge the sample to separate the effect of the teacher-level variable by constructing multilevel structure model.

Finally, this study failed to consider individual variables, for example, previous achievement, as covariates. Previous research has shown that students’ previous achievement influences their perception of parental homework involvement and homework behaviors (Núñez et al., 2017). Besides, high-achieving students perceive more emotional support from teachers (Liao et al., 2016). Therefore, future research in this area should consider and collect potential covariates.

## Data Availability

The datasets generated for this study are available on request to the corresponding author.

## Ethics Statement

This study was carried out in accordance with the recommendations of “Ethical Committee of Central China Normal University” with written informed consent from all subjects. All subjects gave written informed consent in accordance with the Declaration of Helsinki. The protocol was approved by the “Ethical Committee of Central China Normal University.”

## Author Contributions

LG contributed conception and design of the study and was responsible for data collection. YC performed the statistical analysis of the structural equation model. XF and KX contributed to preliminary analyses, manuscript draft, and manuscript revision. SG guided the design of the study, data collection, data analysis, and writing and revision of the manuscript.

## Conflict of Interest Statement

The authors declare that the research was conducted in the absence of any commercial or financial relationships that could be construed as a potential conflict of interest.
